# Required biological time for lung maturation and duration of invasive ventilation: a Korean cohort study of very low birth weight infants

**DOI:** 10.3389/fped.2023.1184832

**Published:** 2023-06-21

**Authors:** Heui Seung Jo, Myoung Nam Lim, Sung-Il Cho

**Affiliations:** ^1^ Department of Pediatrics, Kangwon National University Hospital, Chuncheon, Republic of Korea; ^2^ Biomedical Research Institute, Kangwon National University Hospital, Chuncheon, Republic of Korea; ^3^ Graduate School of Public Health and Institute of Health and Environment, Seoul National University, Seoul, Republic of Korea

**Keywords:** invasive ventilation, lung maturation, bronchopulmonary dysplasia, very low birth weight (VLBW) infant, neonatal network

## Abstract

**Background:**

We investigated the duration of invasive ventilation among very low birth weight (VLBW) infants to evaluate the current minimum time required for lung maturation to breathe without ventilator assistance after preterm birth.

**Methods:**

A total of 14,658 VLBW infants born at ≤32^+6^ weeks between 2013 and 2020 were enrolled. Clinical data were collected from the Korean Neonatal Network, a national prospective cohort registry of VLBW infants from 70 neonatal intensive care units. Differences in the duration of invasive ventilation according to gestational age and birth weight were investigated. Recent trends and changes in assisted ventilation duration and associated perinatal factors between 2017–20 and 2013–16 were compared. Risk factors related to the duration of assisted ventilation were also identified.

**Results:**

The overall duration of invasive ventilation was 16.3 days and the estimated minimum time required corresponded to 30^+4^ weeks of gestation. The median duration of invasive ventilation was 28.0, 13.0, 3.0, and 1.0 days at <26, 26–27, 28–29, and 30–32 weeks of gestation, respectively. In each gestational age group, the estimated minimum weaning points from the assisted ventilator were 29^+5^, 30^+2^, 30^+2^, and 31^+5^ weeks of gestation. The duration of non-invasive ventilation (17.9 vs. 22.5 days) and the incidence of bronchopulmonary dysplasia (28.1% vs. 31.9%) increased in 2017–20 (*n* = 7,221) than in 2013–16 (*n* = 7,437). In contrast, the duration of invasive ventilation and overall survival rate did not change during the periods 2017–20 and 2013–16. Surfactant treatment and air leaks were associated with increased duration of invasive ventilation (inverse hazard ratio 1.50, 95% CI, 1.04–2.15; inverse hazard ratio 1.62, 95% CI, 1.29–2.04). We expressed the incidence proportion of ventilator weaning according to the invasive ventilation duration using Kaplan–Meier survival curves. The slope of the curve slowly decreased as gestational age and birth weight were low and risk factors were present.

**Conclusions:**

This population-based data on invasive ventilation duration among VLBW infants suggest the present limitation of postnatal lung maturation under specific perinatal conditions after preterm birth. Furthermore, this study provides detailed references for designing and/or assessing earlier ventilator weaning protocols and lung protection strategies by comparing populations or neonatal networks.

## Introduction

Bronchopulmonary dysplasia (BPD) is a serious lung disease in premature babies that affects not only the lungs but also growth and development (1–3). With the development of perinatal treatment, the survival rate of premature babies has increased; however, the prevalence rate of BPD has also increased (4–6). After preterm birth, biological time is necessary for lung development and maturation, enabling very preterm infants to survive without respiratory support. Long-term ventilator treatment, which is unavoidable to overcome the anatomical and functional limitations of an immature respiratory system in most cases, can result in tissue injury and inhibit ongoing developmental processes (7, 8). Various respiratory strategies that can minimize lung injury have contributed to the reduction of the duration of invasive ventilation and BPD incidence (6–9). However, the pulmonary structural development of alveolarization is challenging to manipulate using current treatment modalities. In addition, postnatal lung development in preterm infants is not expected to be the same as originally planned during the fetal period. Therefore, predicting the time and natural course of lung maturation is a primary concern for every very low birth weight (VLBW) infant.

The minimum time required for lung maturation sufficient enough to breathe without ventilator assistance after preterm birth is unclear. The duration of ventilator care appears to be mainly affected by the lung’s immaturity. Besides postmenstrual age (PMA), various perinatal conditions, ethnic differences, and epigenetic factors are thought to be related to weaning points. Practically and preferentially, improved pulmonary function results in weaning from assisted ventilation. Spontaneous respiration without a breathing apparatus is a common treatment goal in every neonatal intensive care unit (NICU). Earlier weaning from a mechanical ventilator has many advantages: reduced hospitalization days and costs, lower mortality, and decreased risk of long-term pulmonary sequela, cardiovascular impairment and growth/neurodevelopmental delay (3, 6, 9, 10).

Herein, we aimed to investigate the different duration of assisted ventilation and determine the current PMA limit for ventilator weaning for each gestational age and birth weight group. We analyzed the incidence proportion of invasive ventilator weaning over time for each gestational age and birth weight group of VLBW infants based on the Korean Neonatal Network (KNN) database, a national multicenter prospective registry of VLBW infants in Korea (11). We also compared the current (2017–20) duration of assisted ventilation with that of 2013–16. Furthermore, the effects of associated principal early perinatal risk factors on the duration of assisted ventilation were suggested in the study.

## Materials and methods

### Data collection

Clinical data from 70 NICUs of KNN-participating hospitals were prospectively recorded in the KNN database and analyzed retrospectively in this study. The KNN registry was approved by the institutional review board of each participating hospital, and informed consent was obtained from the parents upon enrollment in the NICUs of KNN-participating hospitals (11). This study protocol was approved by the KNN executive board, and the requested data with decoded hospital and patient information were used to submit the results using these data, which were approved by the Korea Centers for Disease Control and Prevention.

### Study participants

A total of 14,658 VLBW infants born at ≤32^+6^ weeks and registered in the KNN database from January 1, 2013, to December 31, 2020, were included in this study. Infants with major congenital or chromosomal abnormalities were excluded. A total of 7,437 VLBW infants were enrolled in 2013–16 and 7,221 in 2017–20. We identified recent demographical and clinical trends by comparing the rate or distribution of perinatal characteristics among infants born in 2013–16 vs. 2017–20.

Each study variable was defined according to the KNN manual of operation. “Surfactant treatment” was limited to the case of prophylaxis or rescue for respiratory distress syndrome, not for pulmonary hemorrhage or meconium aspiration syndrome. “Air leaks” was limited to a case that required chest tube insertion or needle aspiration. “Massive pulmonary hemorrhage” was severe bleeding enough to cause a cardiovascular collapse or acute respiratory failure. BPD was defined as the need for positive pressure ventilation or oxygen supplementation at 36 weeks PMA. This definition corresponded to the moderate to severe BPD definition from the National Institute of Child Health and Human Development (12, 13).

### Study outcomes

The primary study outcome was the duration of mechanical ventilator support and the estimated weaning time from invasive ventilation according to different gestational age and birth weight groups. Assisted ventilation was defined as any type of invasive or non-invasive positive- pressure ventilation (PPV). Invasive ventilation includes conventional, high-frequency oscillatory ventilation or intermittent PPV through an endotracheal tube. Non-invasive ventilation includes nasal continuous positive airway pressure (CPAP), nasal NIPPV, and high-flow oxygen therapy delivered by a nasal cannula (HFNC) > 2 L/m. When more than one type of ventilator support was used on the same day, the longest-used ventilator was regarded as the main respiratory support mode. We investigated the difference in the duration of invasive and non-invasive ventilation between 2013–16 and 2017–20. We also identified the initial perinatal factors associated with the duration of invasive and non-invasive ventilation.

The secondary outcome was the incidence proportion of ventilator weaning by invasive ventilation duration according to the gestational age (by 2 weeks) and birth weight (by 250 g) among the infants who received invasive ventilator care. The incidence proportion was expressed using the Kaplan–Meier survival curve. We observed that the change in the curve slope or curve shift depends on the presence of specific risk factors.

### Statistical analyses

Data were analyzed using the chi-square test for categorical variables and student’s t-test for continuous variables. The cumulative duration of assisted ventilation was described as the median and interquartile range (IQR). Kaplan–Meier survival curves were used to determine the weaning time of invasive ventilator treatment according to gestational age and birth weight groups. Cox regression analysis was used to identify maternal and initial neonatal factors affecting the required duration of PPV support (invasive and non-invasive). *P *< 0.05 was considered statistically significant. All statistical analyses were performed using the SPSS version 26.0 software (IBM SPSS Statistics, IBM Corporation, Armonk, NY, USA).

## Results

### Maternal and neonatal characteristics

Among the study population of infants (*n* = 14,658), maternal age, the rates of maternal diabetes and hypertension development during pregnancy, and cesarean section increased in the 2017–20 group than in the 2013–16 group (Table 1). The overall maternal age was 33.3 years, 36.1% of infants were born from multiple births, and 85.2% received antenatal steroid therapy between 2013 and 2020.

**Table 1:** Demographics and perinatal characteristics.

Characteristics	Total	2013–16	2017–20	*P*-value
	(*n *= 14,658)	(*n *= 7,437)	(*n *= 7,221)	
Maternal characteristics				
Maternal age (yr)	33.3 ± 4.3	32.9 ± 4.2	33.7 ± 4.4	<0.0001
Multiple gestation	5,285 (36.1)	2,606 (35.0)	2,679 (37.1)	0.009
Maternal diabetes during pregnancy	1,500 (10.2)	636 (8.6)	864 (12.0)	<0.0001
Maternal hypertension during pregnancy	2,984 (20.4)	1,382 (18.6)	1,602 (22.2)	<0.0001
Premature rupture of membrane	5,610 (38.3)	2,872 (38.6)	2,738 (37.9)	0.38
Antenatal steroid therapy	12,483 (85.2)	6,065 (81.6)	6,418 (88.9)	<0.0001
Cesarean section	11,478 (78.3)	5,660 (76.1)	5,818 (80.3)	<0.0001
Neonatal characteristics				
Gestational age (wk)	28^+2 ^± 2^+3^	28^+2 ^± 2^+3^	28^+2 ^± 2^+4^	0.61
<26	2,990 (20.4)	1,526 (20.5)	1,464 (20.3)	0.71
26–27	3,203 (21.9)	1,643 (22.1)	1,560 (21.6)	0.47
28–29	4,338 (29.6)	2,191 (29.5)	2,147 (29.7)	0.72
30–32	4,127 (28.2)	2,077 (27.9)	2,050 (28.4)	0.54
Birth weight (g)	1,050 ± 290	1,050 ± 280	1,050 ± 290	0.14
<750	2,633 (18.0)	1,295 (17.4)	1,338 (18.5)	0.08
750–999	3,535 (24.1)	1,794 (24.1)	1,741 (24.1)	0.99
1,000–1,249	3,996 (27.3)	2,050 (27.6)	1,946 (26.9)	0.40
1,250–1,499	4,494 (30.7)	2,298 (30.9)	2,196 (30.4)	0.52
Males	7,432 (50.7)	3,771 (50.7)	3,661 (50.7)	0.99
Apgar score at 5 min	6.73 ± 1.88	6.67 ± 1.84	6.78 ± 1.91	<0.0001
Surfactant treatment	12,299 (83.9)	6,385 (85.9)	5,914 (81.9)	<0.0001
Air leaks	853 (5.8)	463 (6.2)	390 (5.4)	0.03
Massive pulmonary hemorrhage	955 (6.5)	525 (7.1)	430 (6.0)	0.007
Postnatal steroid therapy	3,744 (25.5)	1,827 (24.6)	1,917 (26.5)	0.006
Duration of hospitalization (d)	69.2 ± 44.0	67.0 ± 42.3	71.5 ± 45.6	<0.0001
Survival rate	12,494 (85.2)	6,318 (85)	6,176 (85.5)	0.44

Values are expressed as numbers (%).

The mean gestational age, birth weight, and the distribution of them did not differ between the 2013–16 and 2017–20 groups (Table 1). Apgar score at 5 min was higher in the 2017–20 group than in the 2013–16 group. The frequencies of surfactant treatment, air leaks, and massive pulmonary hemorrhage were lower in the 2017–20 group. The overall survival rate was 85.2% in the 2013–20 group and was not different between the two groups.

### BPD incidence at 36 weeks of gestation

The overall BPD incidence was 29.9%. The BPD rate increased from 28.1% in 2013–16 to 31.9% in 2017–20 (*P *= 0.0001). Also, the rate of death before 36 weeks of gestation and severe BPD increased in 2017–20 compared to 2013–16 (Table 2). The rate of surviving infants without BPD decreased from 33.5% in 2013–16 to 29.7% in 2017–20.

**Table 2:** Duration of respiratory supports and the incidence of bronchopulmonary dysplasia.

Variables	Total (*n* = 14,658)	2013–16 (*n* = 7,437)	2017–20 (*n* = 7,221)	*P*-value
Duration of respiratory supports (d)				
**A** Invasive ventilation	16.3 ± 28.6	15.9 ± 27.7	16.6 ± 29.5	0.16
**B** Non-invasive ventilation	20.2 ± 21.9	17.9 ± 20.6	22.5 ± 22.9	<0.0001
**C** Supplemental oxygen	6.8 ± 12.6	7.7 ± 13.4	5.9 ± 11.7	<0.0001
** B / A + B**	0.59 ± 0.37	0.55 ± 0.37	0.63 ± 0.36	<0.0001
**A**′ Minimal required maturation time (PMA) of weaning from invasive ventilation (wk)	30^+4 ^± 3^+6^	30^+4 ^± 3^+5^	30^+4 ^± 5^+0^	0.07
BPD at 36^th^ postmenstrual weeks				
Non-BPD	4,659 (31.8)	2,489 (33.5)	2,153 (29.8)	<0.0001
Mild BPD	3,498 (23.9)	1,816 (24.4)	1,682 (23.3)	0.11
Moderate BPD	1,472 (10.0)	809 (10.9)	663 (9.2)	0.001
Severe BPD	2,916 (19.9)	1,278 (17.2)	1,638 (22.7)	<0.0001
Death < 36 weeks	2,113 (14.4)	1,045 (14.1)	1085 (15.0)	0.06
Respiratory support at 36^th^ postmenstrual weeks^a^	(*n* = 12,545)	(*n* = 6,409)	(*n* = 6,136)	
**A** Invasive ventilation	837 (6.7)	407 (6.4)	430 (7.0)	0.14
**B** Non-invasive ventilation	2,302 (18.3)	932 (14.5)	1,370 (22.3)	<0.0001
**C** Supplemental oxygen	3,710 (29.6)	1,774 (27.7)	1,936 (31.6)	<0.0001

Values are expressed as numbers (%); PMA, postmenstrual age; BPD, bronchopulmonary dysplasia; Non-BPD, surviving infants without BPD

^a^ among the surviving 12,545 infants at 36^th^ postmenstrual weeks.

Among the 12,545 infants that survived, the respiratory support status at 36 weeks PMA was as follows: 29.6% needed supplemental oxygen, 18.3% required non-invasive ventilation, and 6.7% needed invasive ventilation. The rate of non-invasive ventilation and the need for supplemental oxygen at 36 weeks PMA were higher in 2017–20 than in 2013–16 (Table 2).

### Duration of respiratory supports

The duration of non-invasive ventilation increased from 17.9 days in 2013–16 to 22.5 days in 2017–20 (Table 2). Also, the proportion of non-invasive ventilation to total PPV (invasive + non-invasive ventilation) was 0.59 ± 0.37, which increased from 0.55 ± 0.37 in 2013–16 to 0.63 ± 0.36 in 2017–20. However, the overall duration of invasive ventilation did not change between 2013–16 and 2017–20. The overall duration of invasive ventilation was 16.3 ± 28.6 days (95% confidence interval, 15.8–16.7), and the median duration (25–75 IQR) was 4.0 (1.0–21.0) days. The median (25–75 IQR) estimated minimal required time for weaning from invasive ventilation, calculated as the corrected gestational age, was 30^+1^ (28^+5^–31^+5^). The mean duration of invasive ventilation according to the gestational age groups was as follows: 35.8 days at <26 weeks of gestation, 23.0 days at 26–27 weeks, 9.2 days at 28–29 weeks, and 4.3 days at 30–32 weeks (Table 3). The median duration (25–75 IQR) of invasive ventilation was 28.0 (9.0–51.0), 13.0 (3.0–31.0), 3.0 (1.0–8.0), and 1.0 (0.0–3.0) days at <26, 26–27, 28–29, and 30–32 weeks of gestation, respectively. With respect to the invasive ventilation duration in the years 2013–16 and 2017–20, there was no difference except in the <26 weeks of gestation group. The median estimated minimal required time for weaning from invasive ventilation was calculated as the corrected gestational age: 28^+6^, 28^+6^, 29^+4^, and 31^+3^ weeks for <26, 26–27, 28–29, and 30–32 weeks, respectively. The mean duration of invasive ventilation according to the birth weight groups was as follows: 35.9 days for <750 g, 23.8 days for 750–999 g, 9.7 days for 1,000–1,249 g, and 4.6 days for 1,250–1,499 g (Table 4). The median duration (25–75 IQR) of invasive ventilation was 27.0 (7.0–51.0), 14.0 (3.0–35.0), 3.0 (1.0–10.0), and 1.0 (0.0–4.0) days for <750 g, 750–999 g, 1,000–1,249 g, and 1,250–1,499 g, respectively. With respect to the invasive ventilation duration in the years 2013–16 and 2017–20, there was no difference except in the <750 g birth weight group. The median estimated minimal required time for weaning from invasive ventilation was 29^+4^, 29^+5^, 29^+6^, and 30^+5^ weeks for <750 g, 750–999 g, 1,000–1,249 g, and 1,250–1,499 g, respectively. Adjusted *p*-values for multiple comparisons between each gestational age or birth weight group are shown in Supplementary Table S1.

**Table 3:** Duration and distribution of assisted invasive ventilation among different gestational age groups.

	Duration of invasive ventilation (d)					Minimal required maturation time^a^ of weaning from invasive ventilation (wk)				
	Mean (SD)	Median	IQR		*p*-value	Mean (SD)	Median	IQR		*p*-value
	95% CI		25th	75th		95% CI		25th	75th	
Gestational age (wk)										
<26 (*n* = 2,990)	35.8 (35.8) 34.6–37.1	28.0	9.0	51.0	0.032	29^+5^ (5^+2^) 29^+4^–29^+6^	28^+6^	26^+0^	32^+0^	0.080
2013–16 (*n* = 1,526)	34.5 (35.0) 32.7–36.2	27.0	8.0	48.0		29^+4^ (5^+1^) 29^+2^–29^+5^	28^+5^	26^+0^	31^+5^	
2017–20 (*n* = 1,464)	37.3 (36.6) 35.4–39.2	30.0	10.0	54.0		29^+6^ (5^+2^) 29^+4^–30^+1^	29^+1^	26^+0^	32^+2^	
26–27 (*n* = 3,203)	23.0 (32.4) 21.9–24.1	13.0	3.0	31.0	0.83	30^+2^ (4^+4^) 30^+1^–30^+3^	28^+6^	27^+5^	31^+2^	0.81
2013–16 (*n* = 1,643)	22.9 (32.3) 21.3–24.5	12.0	3.0	32.0		30^+2^ (4^+4^) 30^+1^–30^+3^	28^+6^	27^+5^	31^+3^	
2017–20 (*n* = 1,560)	23.1 (32.5) 21.5–24.7	13.0	3.0	31.0		30^+2^ (4^+4^) 30^+0^–30^+4^	28^+6^	27^+5^	31^+2^	
28–29 (*n* = 4,338)	9.2 (20.0) 8.6–9.8	3.0	1.0	8.0	0.63	30^+2^ (2^+6^) 30^+1^–30^+3^	29^+4^	29^+0^	30^+2^	0.67
2013–16 (*n* = 2,191)	9.0 (19.5) 8.2–9.9	3.0	1.0	8.0		30^+2^ (2^+6^) 30^+1^–30^+2^	29^+4^	29^+0^	30^+2^	
2017–20 (*n* = 2,147)	9.3 (20.4) 8.5–10.2	2.0	0.0	9.0		30^+2^ (2^+6^) 30^+1^–30^+3^	29^+4^	29^+0^	30^+2^	
30–32 (*n* = 4,127)	4.3 (15.4) 3.8–4.7	1.0	0.0	3.0	0.42	31^+5^ (2^+2^) 31^+4^–31^+6^	31^+3^	30^+5^	32^+2^	0.32
2013–16 (*n* = 2,077)	4.1 (11.6) 3.6–4.6	1.0	0.0	3.0		31^+5^ (1^+6^) 31^+5^–31^+6^	31^+3^	30^+5^	32^+2^	
2017–20 (*n* = 2,050)	4.5 (18.5) 3.7–5.3	0.0	0.0	3.0		31^+6^ (2^+5^) 31^+4^–31^+6^	31^+3^	30^+5^	32^+2^	

^a^ Calculated values (corrected gestational age) based on the ventilation durations and gestational ages; SD, standard deviation; IQR, interquartile range; CI, confidence interval.

**Table 4:** Duration and distribution of assisted invasive ventilation among different birth weight groups.

	Duration of invasive ventilation (d)					Minimal required maturation time^a^ of weaning from invasive ventilation (wk)				
	Mean (SD)	Median	IQR		*p*-value	Mean (SD)	Median	IQR		*p*-value
	95% CI		25th	75th		95% CI		25th	75th	
Birth weight (g)										
<750 (*n* = 2,633)	35.9 (40.3) 34.4–37.5	27.0	7.0	51.0	0.039	30^+3^ (6^+0^) 30^+1^–30^+4^	29^+4^	26^+3^	32^+5^	0.053
2013–16 (*n* = 1,295)	34.3 (38.6) 32.2–36.4	25.0	6.0	48.0		30^+1^ (5^+6^) 29^+6^–30^+3^	29^+2^	26^+3^	32^+2^	
2017–20 (*n* = 1,338)	37.5 (41.9) 35.3–39.8	28.0	8.0	53.0		30^+4^ (6^+2^) 30^+2^–30^+6^	29^+5^	26^+4^	32^+6^	
750–999 (*n* = 3,535)	23.8 (30.4) 22.8–24.8	14.0	3.0	35.0	0.70	30^+4^ (4^+1^) 30^+3^–30^+5^	29^+5^	28^+0^	31^+6^	0.33
2013–16 (*n* = 1,794)	23.6 (31.0) 22.2–25.1	13.0	3.0	35.0		30^+3^ (4^+2^) 30^+2^–30^+5^	29^+5^	27^+6^	31^+6^	
2017–20 (*n* = 1,741)	24.0 (29.8) 22.6–25.4	14.0	2.0	35.0		30^+4^ (4^+1^) 30^+3^–30^+5^	29^+5^	28^+1^	31^+6^	
1,000–1,249 (*n* = 3,996)	9.7 (20.8) 9.1–10.4	3.00	1.0	10.0	0.25	30^+2^ (3^+0^) 30^+2^–30^+3^	29^+6^	28^+5^	31^+2^	0.95
2013–16 (*n* = 2,050)	10.1 (19.8) 9.3–11.0	3.0	1.0	10.0		30^+2^ (2^+6^) 30^+2^–30^+3^	29^+6^	28^+5^	31^+3^	
2017–20 (*n* = 1,946)	9.3 (21.7) 8.4–10.3	2.0	0.0	9.0		30^+2^ (3^+2^) 30^+1^–30^+3^	29^+6^	28^+5^	31^+2^	
1,250–1,499 (*n* = 4,494)	4.6 (12.7) 4.2–5.0	1.0	0.0	4.0	0.31	31^+0^ (2^+0^) 30^+6^–31^+1^	30^+5^	29^+6^	31^+5^	0.75
2013–16 (*n* = 2,298)	4.8 (12.4) 4.3–5.3	2.0	0.0	4.0		30^+6^ (2^+0^) 30^+6^–31^+0^	30^+5^	29^+6^	31^+5^	
2017–20 (*n* = 2,196)	4.4 (13.1) 3.8–4.9	1.0	0.0	4.0		31^+0^ (2^+1^) 30^+6^–31^+1^	30^+5^	29^+6^	31^+6^	

^a^ Calculated values (corrected gestational age) based on the ventilation durations and gestational ages; SD, standard deviation; IQR, interquartile range; CI, confidence interval.

### Perinatal risk factors associated with PPV duration

Maternal and early neonatal risk factors associated with PPV duration were investigated using Cox regression analysis (Table 5). In addition to the gestational age, the presence of surfactant treatment and air leaks increased the risk of prolonged duration of invasive ventilation. In contrast, maternal age, Apgar score at 5 min, and air leaks were associated with the duration of non-invasive ventilation.

**Table 5:** Results of Cox regression analysis of initial perinatal factors associated with duration of invasive and non-invasive ventilation.

Variable	Invasive ventilation		Non-invasive ventilation	
	Inverse hazard ratio (95% CI)	*p-*value	Inverse hazard ratio	*p-*value
Gestational age (per week)	0.86 (0.83–0.89)	<0.001	0.89 (0.87–0.91)	<0.001
Birth weight (per 100 g)	1.00 (0.99–1.00)	0.04	1.00 (0.99–1.00)	0.62
Maternal age	1.00 (0.99–1.02)	0.64	1.01 (1.00–1.02)	0.01
Male	0.91 (0.79–1.04)	0.17	1.02 (0.94–1.11)	0.67
Cesarean section	1.00 (0.83–1.20)	1.00	0.95 (0.85–1.06)	0.34
Multiple gestation	0.93 (0.80–1.07)	0.31	0.96 (0.88–1.05)	0.35
Maternal diabetes during pregnancy	1.01 (0.78–1.30)	0.95	1.09 (0.94–1.26)	0.26
Apgar score at 5min	0.96 (0.92–1.00)	0.06	1.03 (1.01–1.06)	0.01
Surfactant treatment	1.50 (1.04–2.15)	0.03	0.98 (0.83–1.16)	0.84
Air leaks	1.62 (1.29–2.04)	<0.001	0.80 (0.67–0.94)	0.01
Massive pulmonary hemorrhage	1.08 (0.89–1.33)	0.44	0.68 (0.58–0.81)	<0.001

CI, confidence interval.

### Incidence proportion of ventilator weaning by invasive ventilation duration

**Figure 1:**
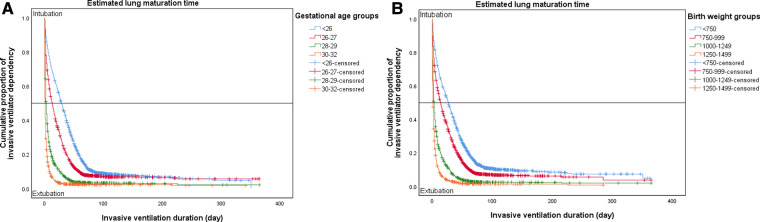
Kaplan–Meier curves showing different cumulative proportions of invasive ventilator dependency based on the duration of invasive ventilator support according to gestational age (**A**) and birth weight (**B**) groups. (**A**) Invasive ventilation log-rank *p*-value < .0001 according to gestational age groups. (**B**) Non-invasive ventilation log-rank *p*-value < .0001 according to birth weight groups.

The Kaplan–Meier model revealed the cumulative proportion of invasive ventilator dependency by invasive ventilation duration for the different gestational age (**A**) and birth weight (**B**) groups (Figure 1). The lower the gestational age or birth weight, the more delayed the successful weaning from invasive ventilation. The weaning point of 50% of the infants was marked in each subgroup.

**Figure 2:**
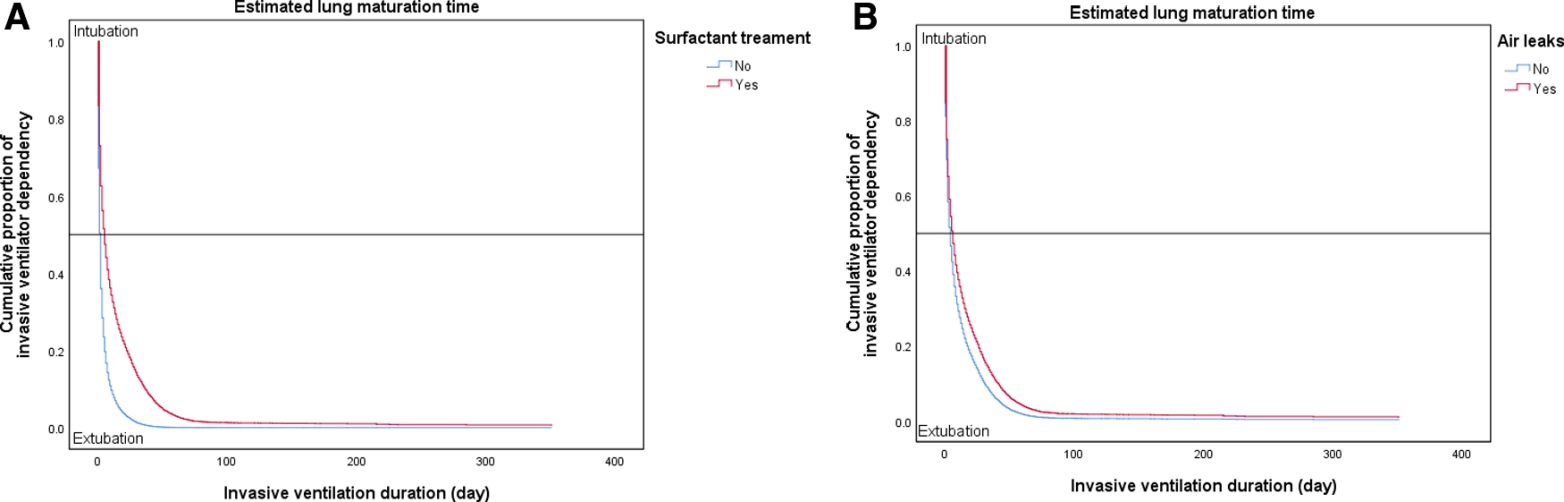
Effects of risk factors on the duration of assisted ventilation. (**A**) Surfactant treatment. (**B**) Air leaks.

The slope of the Kaplan–Meier survival curve slowly decreased in the presence of risk factors such as surfactant treatment or air leaks (Figure 2).

## Discussion

The development of lung function after preterm birth is closely related to gestational age, birth weight, and many other factors that affect the development of alveoli and pulmonary blood vessels (14). Prolonged invasive mechanical ventilation is associated with increased mortality and morbidities related to pulmonary inflammation, developmental delay/arrest, neurodevelopmental impairment, and hospital-acquired infections (2, 8, 10). To avoid tissue injury and inhibition of the ongoing developmental process of premature lungs, various strategies have been developed to minimize invasive mechanical ventilation (4–9). The goal of treatment is to maintain invasive ventilation for only the necessary period of time and switch to non-invasive ventilation as soon as possible. The optimal extubation time implies the theoretical minimum time required for lung maturation enough to breathe without mechanical ventilation. Unfortunately, in the real world, in the clinical field of NICU, it is difficult to predict the exact point of lung maturation sufficient to wean from invasive respiratory support. Population-based PPV duration data are important to accurately predict the optimal timing of extubation and reduce trial and error. Therefore, current qualifying cohort data reflecting the up-to-date treatment strategies are necessary. From the KNN (11), the prospective cohort data of VLBW infants in Korea over 8 years, we assumed the duration of invasive ventilation as the minimal required pulmonary maturation time of weaning from invasive ventilation.

Despite various treatment strategies to prevent BPD development, BPD still remains the most serious chronic lung disease among very preterm infants. Less invasive or non-invasive assisted ventilation, such as nasal CPAP or HFNC, has been applied preferentially to minimize lung injury in VLBW infants (15). Although controversial, recent practices with active application of non-invasive ventilation, rather than invasive ventilation, has contributed to decrease the incidence of BPD and to improve long-term respiratory function (15, 16). According to the KNN data from 2013 to 2014, BPD incidence was 28.9% (17). Compared with the data from 2007 to 2008, the increased incidence of BPD in 2013–14 was probably affected by the increased survival rate of VLBW infants. In this study, the overall BPD incidence between 2013 and 2020 was 29.9%. The incidence of BPD seems to had increased in 2017–20 from 2013–16 (from 28.1% to 31.9%), even with the noticeable increased duration of non-invasive ventilator support (from 17.9 to 22.5 days) (Table 2). Because survival rates were not different between the two groups, it is difficult to conclude that the higher incidence of BPD was a result of more surviving premature infants. Table 1 details recent social and demographic issues, the extremely low birth rate and the increasing proportion of high-risk newborn infants (multiple births, low birth weight, or preterm infants) among the births which are a consequence of advanced maternal age and assisted reproductive technologies (6, 18). The relationship between the incidence of BPD with these demographic changes is a possibility. Further detailed and precise analysis along with discussion are needed to clarify this discrepancy.

In the study population, the mean duration of invasive ventilation was 16.3 days, and the median duration (25–75 IQR) was 4.0 (1.0–21.0) days. The estimated median (25–75 IQR) minimum time required for weaning from invasive ventilation was 30^+1^ (28^+5^–31^+5^) weeks of gestation. The comparison of the duration of invasive ventilation among the gestational age subgroups showed differences except in the 28–29 and 30–32 weeks of gestation groups (Table 3 and Supplementary Table S1). In contrast, the estimated minimum time required for weaning from invasive ventilation showed no differences among the <26, 26–27, and 28–29 weeks of gestation groups. Those in the three groups converged to similar corrected gestational ages: 29^+5^, 30^+2^, 30^+2^ corrected gestational weeks, respectively. These corrected gestational ages are considered a biologically required time for lung maturation sufficient for weaning from invasive ventilation in current NICUs in Korea (Table 3). This information is valuable for determining the optimal ventilator weaning time and reducing trial and error by hasty extubation.

Weisz et al. (19) reported the time of weaning from respiratory support among infants of 23–27 weeks of gestation based on the Canadian Neonatal Network in 2010–17. The corrected gestational ages were 30.4, 30.3, 29.6, 29.0, and 28.3 weeks for infants with 23, 24, 25, 26, and 27 weeks of gestation, respectively. Dassios reported the duration of mechanical ventilation and its association with BPD development using data from the UK (20). Comparison of the duration of invasive ventilation among groups, such as neonatal networks, populations, and hospitals, before and after specific treatments or strategies for lung protection, in the past and present, is a simple and intuitive method for improving quality. It can be used for BPD diagnosis to decrease the conflict between using relatively simple but limited clinical diagnostic methods and complicated diagnostic methods that reflect physiological characteristics (21).

In addition to gestational age, the presence of surfactant treatment and air leakage increased the risk of prolonged duration of invasive ventilation (Table 5 and Figure 2). The Kaplan–Meier survival curve intuitively shows how long the duration of invasive ventilation increases when each risk factor associated with BPD occurrence are present (Figures 1, 2). The ventilator weaning time can be predicted using the steep degree of slope according to the presence or absence of risk factors. The interpretation of risk factors associated with the duration of invasive and non-invasive ventilation requires further consideration of related various demographic phenomena (Table 5).

A limitation of this study is that the start and removal dates of each assisted respiratory ventilator and the exact intubation/extubation dates were not included in the data. Preterm infants born at <26 weeks of gestation or birth weight <750 g required longer invasive ventilation duration in 2017–20 compared to those in 2013–2016. Survival rates according to each gestational age group among 2013–14, 2015–16, 2017–18, and 2019–20 showed no difference including 22, 23, 24, and 25 weeks of gestation subgroups (18). Besides survival rate, more detailed factors and/or phenomena need to be clearly identified which are associated with difference of invasive ventilation duration between 2013 and 16 and 2017–20 among gestational age subgroups <26 weeks of gestation or birth weight <750 g through further analysis.

In conclusion, the present data, a population-based, multicenter cohort study based on the KNN, provided current detailed references on postnatal lung maturation under specific perinatal conditions after preterm birth. VLBW infants require additional biological time for lung maturation and further tailored treatment to overcome immature lung function, especially those with RDS or air leaks. Our results are essential for assessing the therapeutic effects or quality of care for valid comparisons among populations or neonatal networks.

## Data Availability

The original contributions presented in the study are included in the article/**Supplementary Material**, further inquiries can be directed to the corresponding author.
