# Supplementary material for: Required biological time for lung maturation and duration of invasive ventilation: a Korean cohort study of very low birth weight infants
Source: Front Pediatr. 2023 Jun 21;11:1184832. doi: 10.3389/fped.2023.1184832 (PMC10320392; doi:10.3389/fped.2023.1184832)
Supplement: Supplementary file 1 [file Table1.docx]

**TABLE 1. Demographics and perinatal characteristics**

| **Characteristics** | **Total**  **(n=14,658)** | **2013–16**  **(n=7,437)** | **2017–20**  **(n=7,221)** | ***P*-value** |
| --- | --- | --- | --- | --- |
| **Maternal characteristics** | | | | |
| Maternal age (yr) | 33.3 ± 4.3 | 32.9 ± 4.2 | 33.7 ± 4.4 | <0.0001 |
| Multiple gestation | 5,285 (36.1) | 2,606 (35.0) | 2,679 (37.1) | 0.009 |
| Maternal diabetes during pregnancy | 1,500 (10.2) | 636 (8.6) | 864 (12.0) | <0.0001 |
| Maternal hypertension during pregnancy | 2,984 (20.4) | 1,382 (18.6) | 1,602 (22.2) | <0.0001 |
| Premature rupture of membrane | 5,610 (38.3) | 2,872 (38.6) | 2,738 (37.9) | 0.38 |
| Antenatal steroid therapy | 12,483 (85.2) | 6,065 (81.6) | 6,418 (88.9) | <0.0001 |
| Cesarean section | 11,478 (78.3) | 5,660(76.1) | 5,818(80.3) | <0.0001 |
| **Neonatal characteristics** | | | | |
| Gestational age (wk) | 28^+2^ ± 2^+3^ | 28^+2^ ± 2^+3^ | 28^+2^ ± 2^+4^ | 0.61 |
| <26 | 2,990 (20.4) | 1,526 (20.5) | 1,464 (20.3) | 0.71 |
| 26-27 | 3,203 (21.9) | 1,643 (22.1) | 1,560 (21.6) | 0.47 |
| 28-29 | 4,338 (29.6) | 2,191 (29.5) | 2,147 (29.7) | 0.72 |
| 30-32 | 4,127 (28.2) | 2,077 (27.9) | 2,050 (28.4) | 0.54 |
| Birth weight (g) | 1,050 ± 290 | 1,050 ± 280 | 1,050 ± 290 | 0.14 |
| <750 | 2,633 (18.0) | 1,295 (17.4) | 1,338 (18.5) | 0.08 |
| 750-999 | 3,535 (24.1) | 1,794 (24.1) | 1,741 (24.1) | 0.99 |
| 1,000-1,249 | 3,996 (27.3) | 2,050 (27.6) | 1,946 (26.9) | 0.40 |
| 1,250-1,499 | 4,494 (30.7) | 2,298 (30.9) | 2,196 (30.4) | 0.52 |
| Males | 7,432 (50.7) | 3,771 (50.7) | 3,661 (50.7) | 0.99 |
| Apgar score at 5 min | 6.73 ± 1.88 | 6.67 ± 1.84 | 6.78 ± 1.91 | <0.0001 |
| Surfactant treatment | 12,299 (83.9) | 6,385 (85.9) | 5,914 (81.9) | <0.0001 |
| Air leaks | 853 (5.8) | 463 (6.2) | 390 (5.4) | 0.03 |
| Massive pulmonary hemorrhage | 955 (6.5) | 525 (7.1) | 430 (6.0) | 0.007 |
| Postnatal steroid therapy | 3,744 (25.5) | 1,827 (24.6) | 1,917 (26.5) | 0.006 |
| Duration of hospitalization (d) | 69.2 ± 44.0 | 67.0 ± 42.3 | 71.5 ± 45.6 | <0.0001 |
| Survival rate | 12,494 (85.2) | 6,318 (85) | 6,176 (85.5) | 0.44 |

Values are expressed as numbers (%)

**TABLE 2. Duration of respiratory supports and the incidence of bronchopulmonary dysplasia**

| **Variables** | **Total**  **(n=14,658)** | **2013–16**  **(n=7,437)** | **2017–20**  **(n=7,221)** | ***P*-value** |
| --- | --- | --- | --- | --- |
| **Duration of respiratory supports (d)** | | | | |
| **A** Invasive ventilation | 16.3 ± 28.6 | 15.9 ± 27.7 | 16.6 ± 29.5 | 0.16 |
| **B** Non-invasive ventilation | 20.2 ± 21.9 | 17.9 ± 20.6 | 22.5 ± 22.9 | <0.0001 |
| **C** Supplemental oxygen | 6.8 ± 12.6 | 7.7 ±13.4 | 5.9 ± 11.7 | <0.0001 |
| **B / A+B** | 0.59 ± 0.37 | 0.55 ± 0.37 | 0.63 ± 0.36 | <0.0001 |
| **A’** Minimal required maturation time (PMA) of weaning  from invasive ventilation (wk) | 30^+4^ ± 3^+6^ | 30^+4^ ± 3^+5^ | 30^+4^ ± 5^+0^ | 0.07 |
| **BPD at 36^th^ postmenstrual weeks** |  |  |  |  |
| Non-BPD | 4,659 (31.8) | 2,489 (33.5) | 2,153 (29.8) | <0.0001 |
| Mild BPD | 3,498 (23.9) | 1,816 (24.4) | 1,682 (23.3) | 0.11 |
| Moderate BPD | 1,472 (10.0) | 809 (10.9) | 663 (9.2) | 0.001 |
| Severe BPD | 2,916 (19.9) | 1,278 (17.2) | 1,638 (22.7) | <0.0001 |
| Death < 36 weeks | 2,113 (14.4) | 1,045 (14.1) | 1085 (15.0) | 0.06 |
| **Respiratory support at 36^th^ postmenstrual weeks*** | **(n=12,545)** | **(n=6,409)** | **(n=6,136)** |  |
| **A** Invasive ventilation | 837 (6.7) | 407 (6.4) | 430 (7.0) | 0.14 |
| **B** Non-invasive ventilation | 2,302 (18.3) | 932 (14.5) | 1,370 (22.3) | <0.0001 |
| **C** Supplemental oxygen | 3,710 (29.6) | 1,774 (27.7) | 1,936 (31.6) | <0.0001 |

Values are expressed as numbers (%); PMA, postmenstrual age; BPD, bronchopulmonary dysplasia; Non-BPD, surviving infants without BPD; *, among the surviving 12,545 infants at 36^th^ postmenstrual weeks

**TABLE 3. Duration and distribution of assisted invasive ventilation among different gestational age groups**

|  | **Duration of invasive ventilation (d)** | | | |  | **Minimal required maturation time* of**  **weaning from invasive ventilation (wk)** | | | |  |
| --- | --- | --- | --- | --- | --- | --- | --- | --- | --- | --- |
|  | **Mean (SD)** | **Median** | **IQR** | | ***p*-value** | **Mean (SD)** | **Median** | **IQR** | | ***p*-value** |
|  | **95% CI** |  | **25th** | **75th** |  | **95% CI** |  | **25th** | **75th** |  |
| **Gestational age (wk)** | | | | | | | | | |  |
| **<26**  (n=2,990) | 35.8 (35.8)  34.6 - 37.1 | 28.0 | 9.0 | 51.0 |  | 29^+5^ (5^+2^)  29^+4^ - 29^+6^ | 28^+6^ | 26^+0^ | 32^+0^ |  |
| ***2013-16***  (n=1,526) | 34.5 (35.0)  32.7 - 36.2 | 27.0 | 8.0 | 48.0 | **0.032** | 29^+4^ (5^+1^)  29^+2^ - 29^+5^ | 28^+5^ | 26^+0^ | 31^+5^ | **0.080** |
| ***2017-20***  (n=1,464) | 37.3 (36.6)  35.4 - 39.2 | 30.0 | 10.0 | 54.0 |  | 29^+6^ (5^+2^)  29^+4^ - 30^+1^ | 29^+1^ | 26^+0^ | 32^+2^ |  |
| **26-27**  (n=3,203) | 23.0 (32.4)  21.9 - 24.1 | 13.0 | 3.0 | 31.0 |  | 30^+2^ (4^+4^)  30^+1^ - 30^+3^ | 28^+6^ | 27^+5^ | 31^+2^ |  |
| ***2013-16***  (n=1,643) | 22.9 (32.3)  21.3 - 24.5 | 12.0 | 3.0 | 32.0 | **0.83** | 30^+2^ (4^+4^)  30^+1^ - 30^+3^ | 28^+6^ | 27^+5^ | 31^+3^ | **0.81** |
| ***2017-20***  (n=1,560) | 23.1 (32.5)  21.5 - 24.7 | 13.0 | 3.0 | 31.0 |  | 30^+2^ (4^+4^)  30^+0^ - 30^+4^ | 28^+6^ | 27^+5^ | 31^+2^ |  |
| **28-29**  (n=4,338) | 9.2 (20.0)  8.6 - 9.8 | 3.0 | 1.0 | 8.0 |  | 30^+2^ (2^+6^)  30^+1^ - 30^+3^ | 29^+4^ | 29^+0^ | 30^+2^ |  |
| ***2013-16***  (n=2,191) | 9.0 (19.5)  8.2 - 9.9 | 3.0 | 1.0 | 8.0 | **0.63** | 30^+2^ (2^+6^)  30^+1^ - 30^+2^ | 29^+4^ | 29^+0^ | 30^+2^ | **0.67** |
| ***2017-20***  (n=2,147) | 9.3 (20.4)  8.5 - 10.2 | 2.0 | 0.0 | 9.0 |  | 30^+2^ (2^+6^)  30^+1^ - 30^+3^ | 29^+4^ | 29^+0^ | 30^+2^ |  |
| **30-32**  (n=4,127) | 4.3 (15.4)  3.8 - 4.7 | 1.0 | 0.0 | 3.0 |  | 31^+5^ (2^+2^)  31^+4^ - 31^+6^ | 31^+3^ | 30^+5^ | 32^+2^ |  |
| ***2013-16***  (n=2,077) | 4.1 (11.6)  3.6 - 4.6 | 1.0 | 0.0 | 3.0 | **0.42** | 31^+5^ (1^+6^)  31^+5^ - 31^+6^ | 31^+3^ | 30^+5^ | 32^+2^ | **0.32** |
| ***2017-20***  (n=2,050) | 4.5 (18.5)  3.7 - 5.3 | 0.0 | 0.0 | 3.0 |  | 31^+6^ (2^+5^)  31^+4^ - 31^+6^ | 31^+3^ | 30^+5^ | 32^+2^ |  |

* Calculated values (corrected gestational age) based on the ventilation durations and gestational ages; SD, standard deviation; IQR, interquartile range; CI, confidence interval

**TABLE 4. Duration and distribution of assisted invasive ventilation among different birth weight groups**

|  | **Duration of invasive ventilation (d)** | | | |  | **Minimal required maturation time* of**  **weaning from invasive ventilation (wk)** | | | |  |
| --- | --- | --- | --- | --- | --- | --- | --- | --- | --- | --- |
|  | **Mean (SD)** | **Median** | **IQR** | | ***p*-value** | **Mean (SD)** | **Median** | **IQR** | | ***p*-value** |
|  | **95% CI** |  | **25th** | **75th** |  | **95% CI** |  | **25th** | **75th** |  |
| **Birth weight (g)** | | | | | | | | | |  |
| **<750**  (n=2,633) | 35.9 (40.3)  34.4 - 37.5 | 27.0 | 7.0 | 51.0 |  | 30^+3^ (6^+0^)  30^+1^ - 30^+4^ | 29^+4^ | 26^+3^ | 32^+5^ |  |
| ***2013-16***  (n=1,295) | 34.3 (38.6)  32.2 - 36.4 | 25.0 | 6.0 | 48.0 | **0.039** | 30^+1^ (5^+6^)  29^+6^ - 30^+3^ | 29^+2^ | 26^+3^ | 32^+2^ | **0.053** |
| ***2017-20***  (n=1,338) | 37.5 (41.9)  35.3 - 39.8 | 28.0 | 8.0 | 53.0 |  | 30^+4^ (6^+2^)  30^+2^ - 30^+6^ | 29^+5^ | 26^+4^ | 32^+6^ |  |
| **750-999**  (n=3,535) | 23.8 (30.4)  22.8 - 24.8 | 14.0 | 3.0 | 35.0 |  | 30^+4^ (4^+1^)  30^+3^ - 30^+5^ | 29^+5^ | 28^+0^ | 31^+6^ |  |
| ***2013-16***  (n=1,794) | 23.6 (31.0)  22.2 - 25.1 | 13.0 | 3.0 | 35.0 | **0.70** | 30^+3^ (4^+2^)  30^+2^ - 30^+5^ | 29^+5^ | 27^+6^ | 31^+6^ | **0.33** |
| ***2017-20***  (n=1,741) | 24.0 (29.8)  22.6 - 25.4 | 14.0 | 2.0 | 35.0 |  | 30^+4^ (4^+1^)  30^+3^ - 30^+5^ | 29^+5^ | 28^+1^ | 31^+6^ |  |
| **1,000-1,249**  (n=3,996) | 9.7 (20.8)  9.1 - 10.4 | 3.00 | 1.0 | 10.0 |  | 30^+2^ (3^+0^)  30^+2^ - 30^+3^ | 29^+6^ | 28^+5^ | 31^+2^ |  |
| ***2013-16***  (n=2,050) | 10.1 (19.8)  9.3 - 11.0 | 3.0 | 1.0 | 10.0 | **0.25** | 30^+2^ (2^+6^)  30^+2^ - 30^+3^ | 29^+6^ | 28^+5^ | 31^+3^ | **0.95** |
| ***2017-20***  (n=1,946) | 9.3 (21.7)  8.4 - 10.3 | 2.0 | 0.0 | 9.0 |  | 30^+2^ (3^+2^)  30^+1^ - 30^+3^ | 29^+6^ | 28^+5^ | 31^+2^ |  |
| **1,250-1,499**  (n=4,494) | 4.6 (12.7)  4.2 - 5.0 | 1.0 | 0.0 | 4.0 |  | 31^+0^ (2^+0^)  30^+6^ - 31^+1^ | 30^+5^ | 29^+6^ | 31^+5^ |  |
| ***2013-16***  (n=2,298) | 4.8 (12.4)  4.3 - 5.3 | 2.0 | 0.0 | 4.0 | **0.31** | 30^+6^ (2^+0^)  30^+6^ - 31^+0^ | 30^+5^ | 29^+6^ | 31^+5^ | **0.75** |
| ***2017-20***  (n=2,196) | 4.4 (13.1)  3.8 - 4.9 | 1.0 | 0.0 | 4.0 |  | 31^+0^ (2^+1^)  30^+6^ - 31^+1^ | 30^+5^ | 29^+6^ | 31^+6^ |  |

* Calculated values (corrected gestational age) based on the ventilation durations and gestational ages; SD, standard deviation; IQR, interquartile range; CI, confidence interval

**Supplementary Table 1. Adjusted *p*-values for multiple comparisons among each subgroups of Table 4 using Bonferroni correction**

|  | **Duration of invasive ventilation (d)** | | | |  | **Minimal required maturation time* of**  **weaning from invasive ventilation (wk)** | | | |
| --- | --- | --- | --- | --- | --- | --- | --- | --- | --- |
| **Gestational age (wk)** | | | | | | | | | |
|  | **<26** | **26-27** | **28-29** | **30-32** |  | **<26** | **26-27** | **28-29** | **30-32** |
| **<26** | - | <0.001 | <0.001 | <0.001 | **<26** | - | 0.30 | 0.28 | 0.001 |
| **26-27** | <0.001 | - | 0.004 | 0.003 | **26-27** | 0.30 | - | 0.94 | 0.04 |
| **28-29** | <0.001 | 0.004 | - | 0.73 | **28-29** | 0.28 | 0.94 | - | 0.02 |
| **30-32** | <0.001 | 0.003 | 0.73 | - | **30-32** | 0.001 | 0.04 | 0.02 | - |
|  | | | | | | | | | |
| **Birth weight (g)** | | | | | | | | | |
|  | **<750** | **750-999** | **1,000-1,249** | **1,250-1,499** |  | **<750** | **750-999** | **1,000-1,249** | **1,250-1,499** |
| **<750** | - | <0.001 | <0.001 | <0.001 | **<750** | - | 0.02 | 0.99 | 0.97 |
| **750-999** | <0.001 | - | 0.84 | 0.56 | **750-999** | 0.02 | - | 0.003 | 0.001 |
| **1,000-1,249** | <0.001 | 0.84 | - | 0.27 | **1,000-1,249** | 0.99 | 0.003 | - | 0.98 |
| **1,250-1,499** | <0.001 | 0.56 | 0.27 | - | **1,250-1,499** | 0.97 | 0.001 | 0.98 | - |

**TABLE 5. Results of Cox regression analysis of initial perinatal factors associated with duration of invasive and non-invasive ventilation**

| Variable | **Invasive ventilation** | | **Non-invasive ventilation** | |
| --- | --- | --- | --- | --- |
|  | Inverse hazard ratio  (95% CI) | *p-*value | Inverse hazard ratio  (95% CI) | *p-*value |
| Gestational age (per week) | 0.86 (0.83 - 0.89) | <0.001 | 0.89 (0.87 - 0.91) | <0.001 |
| Birth weight (per 100g) | 1.00 (0.99 - 1.00) | 0.04 | 1.00 (0.99 - 1.00) | 0.62 |
| Maternal age | 1.00 (0.99 - 1.02) | 0.64 | 1.01 (1.00 - 1.02) | 0.01 |
| Male | 0.91 (0.79 - 1.04) | 0.17 | 1.02 (0.94 - 1.11) | 0.67 |
| Cesarean section | 1.00 (0.83 - 1.20) | 1.00 | 0.95 (0.85 - 1.06) | 0.34 |
| Multiple gestation | 0.93 (0.80 - 1.07) | 0.31 | 0.96 (0.88 - 1.05) | 0.35 |
| Maternal diabetes during pregnancy | 1.01 (0.78 - 1.30) | 0.95 | 1.09 (0.94 - 1.26) | 0.26 |
| Apgar score at 5min | 0.96 (0.92 - 1.00) | 0.06 | 1.03 (1.01 - 1.06) | 0.01 |
| Surfactant treatment | 1.50 (1.04 - 2.15) | 0.03 | 0.98 (083 - 1.16) | 0.84 |
| Air leaks | 1.62 (1.29 - 2.04) | <0.001 | 0.80 (0.67 - 0.94) | 0.01 |
| Massive pulmonary hemorrhage | 1.08 (0.89 - 1.33) | 0.44 | 0.68 (0.58 - 0.81) | <0.001 |

CI, confidence interval
